# Chiari type I malformation of infants and toddlers

**DOI:** 10.1007/s00381-017-3712-7

**Published:** 2018-02-02

**Authors:** Gordan Grahovac, Tatiana Pundy, Tadanori Tomita

**Affiliations:** Division of Pediatric Neurosurgery, Ann & Robert H. Lurie Children’s Hospital of Chicago, Northwestern University Feinberg School of Medicine, 225 E. Chicago Avenue, Chicago, IL 60611 USA

**Keywords:** Chiari type 1 malformation, Infants, Posterior fossa decompression

## Abstract

**Objectives:**

Chiari I malformation has been a well-recognized clinical entity; however, its occurrence among infants and toddlers is unusual. Their clinical presentations may be different from other age groups due to their lack of effective verbal communication. The authors analyze their personal series of patients focusing on symptomatology and MRI characteristics. Treatment methods, results, and outcome are analyzed in order to identify appropriate surgical management among infants and toddlers with Chiari I malformation.

**Methods:**

The authors retrospectively reviewed 16 patients who were diagnosed and surgically treated between 2007 and 2014 during the first 3 years of life with minimum follow-up of 3 years. We focused on the presenting symptoms, magnetic resonance imaging findings, and surgical techniques used for posterior fossa decompression (PFD) and their postoperative outcome.

**Results:**

Twelve patients (75%) presented with signs of headaches such as irritability, inconsolable crying, head grabbing, and/or arching back. Ten patients (62.5%) presented with oropharyngeal and/or respiratory symptoms such as emesis, choking, gagging, snoring, sleep apnea, breathing pause, and/or vocal cord palsy. Only one patient had segmental cervical hydromyelia. At the first surgery, ten patients had PFD with dural scoring (Type 1 procedure), while six others had PFD with duraplasty (Type 2 procedure) with thermal reduction of the cerebellar tonsils in four. Following the first operation, all initially had varying degrees of symptomatic improvement; however, seven patients subsequently had symptomatic recurrence. Persistent crowding at the PFD site on the postoperative imaging indicated greater risk of recurrences in both Type 1 procedure and Type 2 procedure groups. Of seven patients who needed a second operation, fivewere after Type 1 procedure and the two were after Type 2 procedure. The difference of recurrence rates between these two groups is not significant. CSF-related complications occurred in 4 out of 11 patients who had Type 2 procedure (one after primary decompression and three after the second decompression for recurrence).

**Conclusion:**

Young patients lacking effective verbal communication often present their Chiari I malformation differently from olderage groups. Behavioral changes indicative of headaches/irritability and oropharyngeal/respiratory symptoms are the primary presenting symptoms. The recurrence rate tends to be higher among the patients after Type 1 procedure (particularly those younger than 18 months) than after Type 2 procedure. We observed that duraplasty at primary or at redo PFD provides for better decompression and long-term outcome. However, one should keep it in mind that there is risk of CSF-related complications following duraplasty, particularly higher tendency after redo PFD.

## Introduction

Chiari type I malformation is one of the four hindbrain malformations first described in pathological studies by Hans Chiari in 1891 [1]. Chiari type I malformation is now frequently found incidentally due to wide spread use of magnetic resonance imaging (MRI) while investigating different clinical scenarios such as head trauma, epilepsy, developmental delays, headache, and migraines. Chiari I malformation is a morphological hindbrain malformation which may evolve into a symptomatic condition as child grows [2]. Occipital headaches are the most common signs and symptoms of Chiari I malformation and can be induced during Valsalva maneuver such as coughing, sneezing, and laughing, or during exercise. Other symptoms include brainstem and spinal cord dysfunction due to direct compression or hydromyelia causing upper or lower motor neuron dysfunction resulting in weakness, spasticity, and ataxia. There are sensory deficits, respiratory dysfunction, and lower cranial nerve deficits causing dysarthria, dysphagia, soft palate weakness, diminished gag reflex, and vocal cord deficits [3–7]. The development of scoliosis associated with hydromyelia is also frequently described with Chiari type I malformations [8–11].

Most of the literature concerning pediatric Chiari malformation focuses on elementary-aged children and teenagers. According to recent publications US NSQIP-Pediatric database, and Healthcare Cost and Utilization Project State Inpatient Database, the majority of pediatric patients with Chiari I malformation were aged 5–14 and 6–15 years, respectively [12, 13]. Reports of Chiari I malformation among extremely young population are rare and their presentation may differ from older children [14]. Two other publications, both from the same institution, have focused their research on Chiari I patient groups younger than 6 years of age [15, 16]. According to these reports, common presentation was oropharyngeal dysfunction such as snoring, coughing, dysphagia, sleep apnea, and choking which occurred in nearly half of these young patients (51.3%). In their series, 77.8% of patients of 0–2-year group presented with oropharyngeal dysfunction, while only 38.1% of 3–5-year group did [15]. On the other hand, the 0–2-year group had lower frequency of headaches (33.3%) compared with the olderage group (57.1%).

Among infants and toddlers who lack effective verbal communication, their symptoms differ from their older counterparts. Their presenting symptoms are irritability, inconsolable cry, arching back, or gagging [17].

The authors analyze their personal series of patients younger than 3 years old focusing on symptomatology and MRI characters. Treatment methods, results, and outcome are analyzed in order to identify appropriate surgical management among infants and toddlers without adequate verbal ability.

## Materials and methods

A retrospective study was done on 16 patients who underwent surgical decompression of Chiari I malformation at the Ann & Robert H. Lurie Children’s Hospital of Chicago during the first 3 years of life from June 2007 to November 2014. The study was approved by Lurie Children’s IRB No. 2012-15083. Patients’ age at the time of surgical intervention ranged from 5 to 35 months. Follow-up after surgery ranged from 3 to 10 years, with a mean of 5.6 years. Infants and toddlers who did not have surgical intervention were not included.

Preoperative and postoperative MRIs were reviewed for all the 16 patients. Midsagittal brain MRI sequence was used for radiographic measurement. We measured the caudal extent of the tonsils below the McRae line. The basal angle of skull base was measured using modified MR imaging technique described by Koenigsberg et al. [18]. The Grabb classification of the pB-C2 line (drawn perpendicular to a line drawn between the basion and the posterior aspect of the C2 vertebral body, at the most posterior extent of the odontoid process at the dural interface) is attained to see the degree of ventral compression [19, 20].

The posterior fossa decompression (PFD) and upper cervical laminectomy were performed as previously described by Navarro et al. from our institution [21]. Two different types of surgical procedure were used to treat these patients. Type 1 consisted of PFD and partial thickness durotomy (dural scoring). Type 2 consisted of PFD and opening of the dura with dural patch grafting (duraplasty) using Durepair® (Medtronics) with or without tonsillar thermal reduction with a bipolar cautery. Dural closure was done under a surgical microscope. Intraoperative ultrasonography (US) was used to evaluate intradural structures following bone decompression. When US showed bone decompression alone was not sufficient because of lack of CSF space dorsal to the herniated cerebellar tonsils, Type 2 procedure was performed.

Outcome following surgery was evaluated for symptomatic and MRI improvements. Follow-up MRI was obtained within the next 3–6 months, then annually for 3 years or at the time of symptom development. Complications, recurrences, and additional surgical procedures were also reviewed during the follow-up time.

## Results

Sixteen patients, eight females and eight males, underwent surgical decompression for Chiari I malformation. At the initial surgery, four patients were younger than 12 months, six patients between 12 and 23 month, and six patients in 24 to 36 months. Clinical presentation, surgical procedure, and outcome are shown on Table 1. All had isolated Chiari I malformation, but one, Case 9, had neurofibromatosis type 1.

**Table 1:** Clinicalsummary of 16 infants and toddlers with Chiari I malformation

Case	Age	Sex	Symptoms	Tonsillar ectopia		1st operationtechnique	Postop outcome (after 1st op)		Recurrence symptoms	Time to 2nd operation (months)	Reoperationtechnique	Postop outcome (2nd op)	
	(months)			Level	Distance(mm)		Symptoms	MR (3–6 months)				Symptom	sMR
1	6	M	Inconsolable cry with arching back, nighttime waking	C1	6	Type 1	Minor	Unchanged	Grabbing occiput, nighttime waking	22	Type 2*	Improved	Improved
2	7	F	Stridor (left vocal cord paresis), spasticity	C1	5	Type 1	Temporary	Unchanged	Gagging,vocal cord paresis	3	Type 2* + PMC	Improved	Improved
3	11	F	Nighttime waking with coughing/snoring, inconsolable cry	C1–2	10	Type 1	Improved	Unchanged	Gagging, snoring	35	Type 2*	Improved	Improved
4	17	f	Head grabbing, coughing bout or cries, nighttime waking	C2	12	Type 1	Temporary	Unchanged	Nighttime waking	24	Type 2* + PMC	Improved	Improved
5	19	M	Emesis with weight loss, occipital grabbing, irritability	C1	4	Type 1	Improved	Improved	–	–	–		
6	21	M	Breath holding spells, seizures, irritability	C1–2	10	Type 1	Improved	Min. improved	–	–	–		
7	25	M	Bilateral esotropia	C2	7	Type 1	Temporary	Unchanged	Headaches, emesis,hydrocephalus	7	Type 2* + PMC	Improved	Improved
8	29	M	Crossing eyes with irritability, sleep apnea, holding back of head	C2	8	Type 1	Improved	Unchanged	–	–	–		
9	33	F	NF1. holding head,l emesis	C1–2	12	Type 1	Improved	Unchanged	–	–	–		
10	35	F	Failure to thrive, irritability, inconsolable cry with arching back	C1	8	Type 1	Improved	Improved	–	–	–		
11	11	F	Increased head size	C2	11	Type 2*	Improved	Improved	–	–	–		
12	19	M	Holding head with screaming	C1	7	Type 2	Improved	Improved	–	–	–		
13	19	F	Failure to thrive, poor gain of weight	C2–3	16	Type 2*	Improved	Improved	–	–	–		
14	23	M	Sleep apnea, inconsolable cry with arching back	C2–3	10	Type 2* + PMC	Temporary	Unchanged	Head grabbing, cough, motor weakness	8	Type 2*	Improved	Improved
15	27	M	Developmental delay, nighttime waking, head rubbing	C1–2	13.5	Type 2*	Improved	Improved	–	–	–		
16	30	F	Failure to thrive, holding head expressing pain,gait ataxia	C1–2	8	Type 2	Improved	Min. improved	Headache, nighttime waking	15	Type 1	Improved	Improved

Type 2* indicates “duraplasty with tonsillar reduction”

*PMC*pseudomeningocele

### Presenting symptoms

Twelve patients (75%) presented with signs of headaches such as irritability, inconsolable cries, nighttime waking, head grabbing, and/or back arching. Ten patients (62.5%) presented with oropharyngeal symptoms such as emesis, choking, gagging, dysphagia, snoring, sleep apnea, or breathing pause. Long tract signs were noted in two patients: one with spasticity and another with gait instability.

### Neuroimaging

We reviewed pre- and postoperative MRI studies of all the 16 patients. The tonsillar descents below the foramen magnum ranged from 4 to 16 mm with mean of 9.8 mm. The position of the tonsillar tips was above or at C1 level in five, at C1–2 level in five, at C2 level in four, and at C2–3 level in two. The mean basal angle in all age groups was 122.5° (range 111.9°–134°). The mean pB-C2 line in all age groups was 6.33 mm (range 4.5–9.2 mm).

Hydrocephalus was noted in two patients (Cases 11 and 13). Hydromyelia was seen on presentation in one patient (Case 7), which was small and located at C2 level. Three others (Cases 4, 9, and 12) had T2/FLAIR hyper-intensity at C2–3. None of the patients had scoliosis at the time of presentation.

### Surgical decompression

All the children in this series underwent PFD with C1 posterior arch removal. In two patients, additional C2 laminectomy was also performed. At the PFD, the upper edge of craniectomy was below the superior occipital line.

At the first surgery, Type 1 procedure (dural scoring) was done in ten cases and Type 2 procedure (duraplasty) was performed in six. Three patients (Cases 11, 13, and 14) with severe tonsillar descent at C2 or below underwent Type 2 procedure. Other three patients who were considered to be inadequately decompressed because of lack of CSF space dorsal to the herniated cerebellar tonsils by intraoperative US underwent Type 2 procedure. Amongsix patients with Type 2 procedure, four also received thermal tonsillar reductions.

### Management of hydrocephalus

Two patients with hydrocephalus at diagnosis had a CSF diversion at the time of PFD: placement of external ventricular drainage (EVD) in Case 11 and endoscopic third ventriculostomy (ETV) in Case 13. Both had a Type 2 procedure.

### Postoperative course

Only one of six patients (Case 14) who had Type 2 procedure developed CSF leak from the wound and required reoperation 5 days postoperatively. Another patient (Case 11) was treated for persistent hydrocephalus after Type 2 procedure with EVD and then required ETV. Another infant with hydrocephalus (Case 13) had a concurrent ETV and Type 2 procedure, but subsequently needed VP shunt placement.

Presenting symptoms were improved in all the patients after the initial surgical intervention though the improvement was minimal or temporary in five patients.

### Postoperative MR findings

**Fig. 1:**
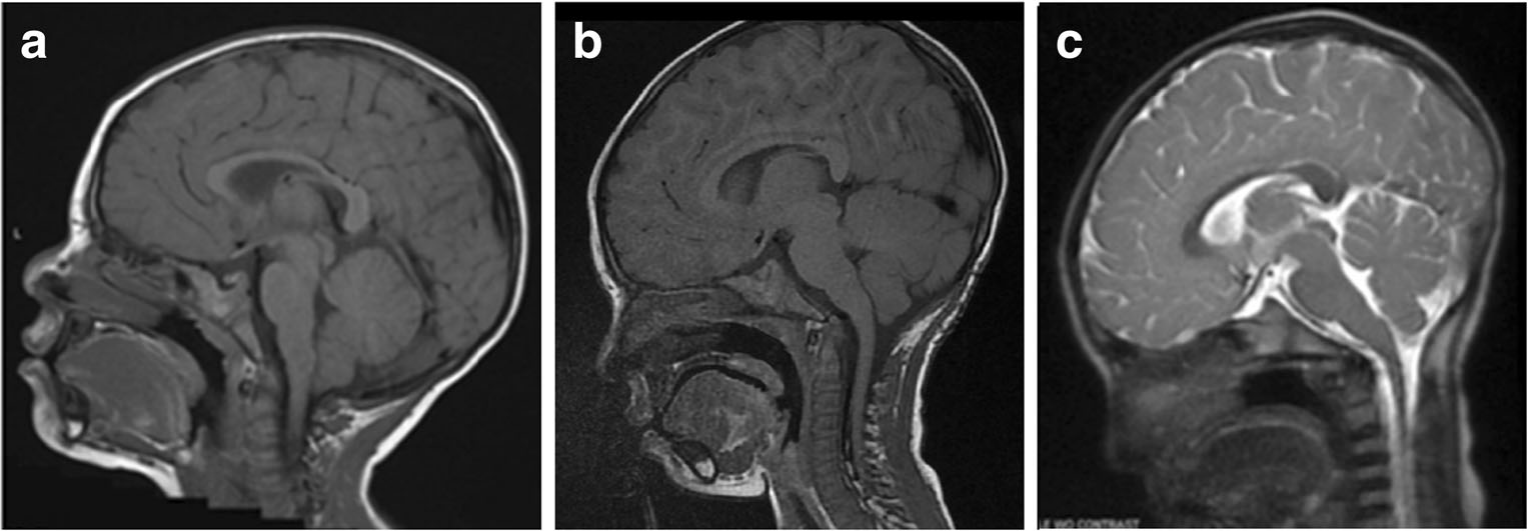
A 20-month-old boy (Case 5) with a 3-month history of increasing emesis and grabbing his occiput. Preoperative midsagittal T1-weighted MRI (A) showed Chiari I malformation with tonsils herniating up to above the C1. He had a posterior fossa decompression with dural scoring (Type 1 procedure). Postoperatively, all symptoms resolved, andwith improved decompression site shown on midsagittal T1-weighted MR (B) obtained 4 months postoperatively. A 3-year postoperative MR showed spontaneous improvement of the decompression site (C).

On the initial postoperative MRI following Type 1 procedures, seven patients had little or no changes of the low set tonsils and tightness at the craniocervical junction. Another three showed mild CSF space expansion. Of the latter, two patients (Cases 5 and 10) showed late improvements in the tonsillar herniation and expanding CSF space at the craniocervical junction, which were noted 2.5 and 3 years postoperatively (Fig. 1). Another patient (Case 8) had no initial MRI improvement, but subsequent MRI showed gradual expansion of the CSF space after 4 years, and further improvement was noted 9 years later. Of seven patients with no or little improvement in tonsillar ectopia and crowded craniocervical junction, five patients subsequently developed recurrent symptoms, needing a reoperation.

In the group of Type 2 procedure, the craniocervical junction crowding improved in all but one (Case 14) who subsequently had postoperative pseudomeningocele which was repaired shortly after the initial surgery. Case 16 had later a symptomatic recurrence and needed a reoperation. Segmental hydromyelia in Case 7 and T2/FLAIR intensity noted preoperatively in Cases 4, 9, and 12 resolved after the decompression.

Six out of eight patients who did not show improvements on postoperative MRI subsequently developed symptomatic recurrence, while only one of eight patients who had improvement on MRI had recurrence (chi-square: *p* = 0.0027). In both Type 1 procedure and Type 2 procedure groups, postoperative MRI appeared to predict the outcome.

### Recurrences

Seven patients underwent a second decompression procedure due to symptomatic recurrence:five patients (50%) after Type 1 procedure and the two patients (33.3%) after Type 2 procedures. Four of five recurrences after Type 1 procedure occurred in patients younger than 18 months. There was a tendency of less recurrence after the Type 2 procedure but no statistical difference (chi-square: *p* = 0.515305).

**Fig. 2:**
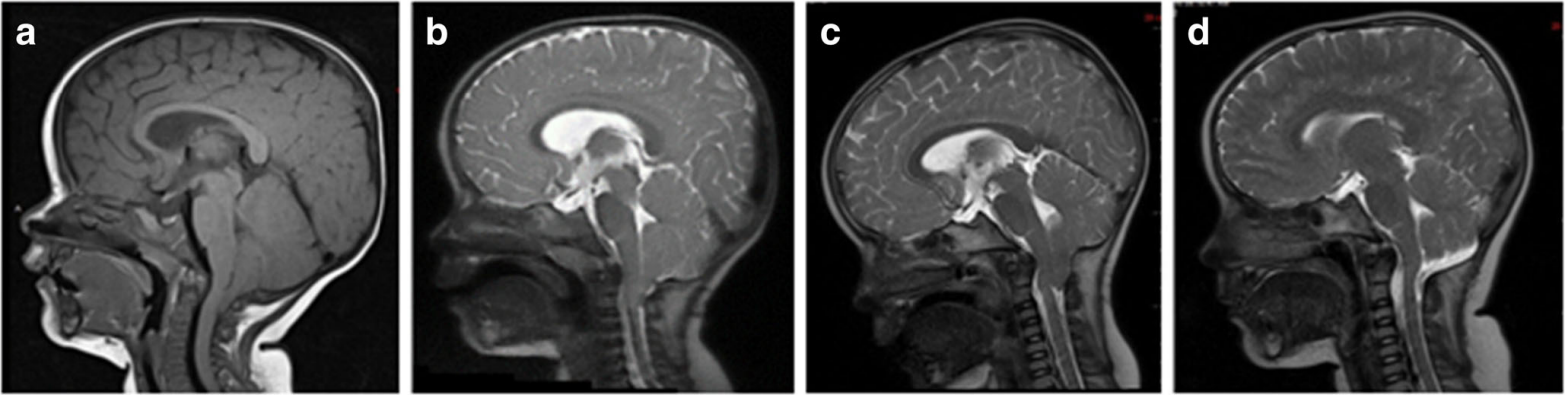
An 11-month-old girl (Case 3) presented initially with increasing snoring and coughing during the sleep, and more recently, the patient showed irritability with holding the back of her head. Midsagittal T1-weighted MRI (A) showed Chiari I malformation with tonsils herniating up to above portion of the C2 lamina. There is an abnormal intensity in the upper cervical cord. She underwent posterior fossa decompression and dural scoring(Type 1 procedure) and postoperatively all her symptoms resolved. Midsagittal fast T2-weighted MR (B), 3 months after the decompression, showing little improvement in the decompressed foramen magnum. Three years later, she developed grabbing occiput, nighttime waking, and snoring. Midsagittal fast T2-weighted MR (C) showing a tight posterior fossa and upper cervical space with medullary compression. A reoperation with tonsillar thermal reduction and dural patch graft was performed. Her snoring promptly resolved and the latest MR done 2 years after the second operation showed a good decompression (D)

Additional PFD was done in five patients (Cases 1–4 and 7) following Type 1 procedure, in 3, 7, 22, 24, and 35 months, respectively. Notably, four out of five recurrences occurred among patients whose age at Type 1 procedure was less than 19 months. Of those, Cases 3 and 4, who had reoperation 24 and 35 months later, narrowing of the craniectomy was observed due to re-ossification (Fig. 2). All five patients had duraplasty with tonsillar reduction at the second surgery; three of them, Cases 2, 4, and 7, developed postoperative pseudomeningocele requiring surgical repair. The pseudomeningocele was repaired by re-exploration in Case 2. Case 4 who developed pseudomeningocele and acute hydrocephalus was treated with temporary EVD. Case 7 had external CSF leak, resulting in meningitis and subsequent hydrocephalus which was treated with wound repair and VP shunt. All these became asymptomatic. In this series, there is a higher risk of CSF-related complications after reoperation with duraplasty following Type 1 procedure, three out of five, than after primary Type 2 procedures, one out of six, albeit no statistical difference (*p* = 0.133614).

**Fig. 3:**
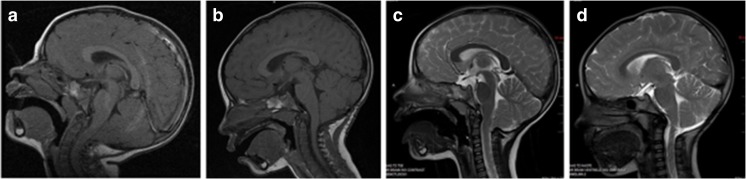
A 30-month-old girl (Case 16) who presented with increasing irritability, difficulty to swallow, and tendency to fall. In past several days, the patient woke up screaming. Midsagittal T1-weighted MR showed a Chiari I malformation with tight posterior fossa with the tonsils below the C1 level (A). A posterior decompression with dural patch grafting was done. Postoperative MR done 3 months later showed increased subarachnoid space but the position of tonsillar tips remained unchanged (B). Her symptoms resolved shortly after the surgery. However, 9 month later, she started to experience increasing headaches. Midsagittal fast T2-weighted MR done 13 months postoperatively showed the tonsils descending at the level of the previous C1 and C2 junction with epidural tissue growth causing constriction at that level (C). At reoperation, a thick fibrous band at C1 level was removed. An expansion of thecal sac was observed without opening the dura. MR done 2 years after the second surgery showed excellent decompression and she was asymptomatic (D).

One child, Case 16, needed a second operation 15 months following Type 2 procedure. At the second operation, a thick epidural fibrous band was noted at the former decompression site which was released without opening the dura and symptoms resolved (Fig. 3). Another patient, Case 14, re-developed on and off gagging, occipital headaches, and coughing episodes, and 8 month later, developed acute motor weakness following coughing spells needing another operation with further tonsillar reduction and PFD.

None of the patients showed further recurrent symptoms since their last surgical intervention during the follow-up period.

## Discussion

The current literature is scarce regarding the surgical treatment of patients with Chiari type I malformation specific to children younger than 3 years of age. In such young populations, symptoms of Chiari type I malformations are different from those observed in older patients partly due to lack of adequate verbal language [14–16]. The incidence of headache as a presenting symptom was lower than in adolescents and young patients [15]. Among young patients, behavioral changes such as unexplainable irritability, inconsolable crying, head grabbing, or opisthotonos are the common symptoms indicative of headaches. Oropharyngeal symptoms and respiratory symptoms such as dysphagia, emesis, choking, gagging, snoring, sleep apnea, breath holding, and/or vocal cord palsy were second most common presenting symptoms, noted in 62.5% of our patients. These oropharyngeal dysfunctions including reflux, dysphagia, snoring, and episodes of apnea occur more often in the younger age group [15]. Albert et al. noted that frequency of syrinx was significantly (*p* = 0.0002) lower among the 0–2-year group (27.8%) than the 3–5-year group (85.7%) [15]. These authors reported that scoliosis was noted in 16.7% in the 0–2-year age group [15]. However, our results indicate they are rare; only one syrinx and no scoliosis in our series.

One of the main causes of Chiari I malformation is underdevelopment of the mesodermal occipital somite that leads to the posterior fossa being of small volume and thus causes abnormal CSF flow at the level of skull base and foramen magnum [22]. Surgical treatment is primarily focused on restoring the normal CSF flow at the level of the foramen magnum with enlargement of the posterior fossa and upper cervical subarachnoid space. The primary treatment of symptomatic Chiari I malformations is PFD. According to Albert et al., a majority of their patients underwent the duraplasty with cervical fascia with or without an intradural exploration to identify the arachnoid web or veil impairment at the level of foramen Magendie [15]. In our study, all of our patients underwent PFD with C1 laminectomy (additional C2 laminectomy in two patients). All the patients underwent the partial thickness durotomy or dural scoring (Type 1 procedure) and the rest of the patients underwent the duraplasty (Type 2 procedure).

Intraoperative US is a useful tool to assess the degree of decompression. Improved tonsillar pulsation and increased subarachnoid space at the retrocerebellar and upper cervical locations are considered to be the indication of the successful decompression. Narenthiran et al. reported that there were no difference between dural decompression group and bony decompression group regarding postoperative improvement in the syrinx based on intraoperative US findings [23]. However, in our ten patients who underwent Type 1 procedure and were considered to have adequate decompression by intraoperative US, five ultimately developed recurrence following temporary symptomatic improvement.

Bone decompression alone vs. duraplasty for effective Chiari decompression remains controversial [21]. The former poses a concern of inadequate decompression, while the latter poses a concern of CSF-related complications such as chemical meningitis, CSF pseudomeningocele, CSF leak, subdural hygroma, and hydrocephalus. In the literature, intradural manipulations can have complication rates up to 42% compared with complication rate of only of 10% in procedures without opening of the dura [8, 24, 25]. Impaired CSF circulation can cause CSF leak and development of hydrocephalus or subdural hygroma [26, 27]. In our series, out of seven who required additional procedure for treatment of the recurrent symptoms, five were after Type 1 procedure and two after Type2. There is no statistical difference between the two groups for recurrence rate (chi-square: *p*value= 0.515305).

In our series, one of six patients who had Type 2 procedure developed postoperative pseudomeningocele. On the other hand, three out of five patients, who had a second PFD with duraplasty after initial Type 1 procedure, developed postoperative pseudomeningocele; two of them resulted in hydrocephalus. It is not clear as to why the incidence of pseudomeningocele was higher after duraplasty at redo following the Type 1 procedure. Perhaps previous dural scoring may have caused weaker integrity of regenerated dura. Thus, CSF-related complication occurred in 4 out of 11 patients who had duraplasty in our series (one after primary decompression and three after the second decompression).

We performed aggressive scoring of the dura at Type 1 procedure in ten patients, assuming infantile dura is more elastic. However, effective dural scoring or stripping external dural layer is possible for the spinal dura. In this series, aggressive durotomy leaving very thin layer of the dura was carried out, which made cerebellar tonsils visible in the expanded spinal subarachnoid space through thin covering under a microscope. Posterior fossa dura, particularly of infants, is too thin to separate the outer layer from the inner layer. However, the posterior fossa dura is more expansible than the spinal dura. Following Type 1 procedure, postoperative MR showed persistent tonsillar descent and tight craniocervical junction in six and minimum improvements in two. However, a significant expansion of the subarachnoid space at the decompression site was noted in two patients after 2.5 and 3 years, respectively. Thus, a delayed dural expansion may occur years after the bony decompression with dural scoring.

The radiological measurement of the craniocervical junctions in pediatric Chiari I malformations may be useful but its clinical significance still has been unknown. The literature indicates that the posteriorly inclined odontoid process has been demonstrated in 23.3–84% cases depending on radiological methodology [5, 19, 28].

Little is known about the clinical value of pB-C2 line in the patients younger than age 3. According to the Tubbs classification, anterior spinal canal encroachment is categorized as low (< 6 mm), medium (6–9 mm), and high (> 9 mm) [19]. In our series, pB-C2 line ranged from 4.5 to 9.2 mm with mean of 6.33 mm; thus, a majority of patients could be categorized with the medium grade. Only two patients (Cases 13 and 14) in our series had anterior spinal canal encroachment categorized as high grade, with the pB-C2 greater than 9 mm. It was described in the literature that patients with pB-C2 line greater than 9 mm are more likely to need anterior cervical decompression or occipitocervical fusion [20, 29]. Our patient with pB-C2 line longer than 9 mm did not require anterior decompression, although the length of follow-up was not long enough (3 and 5 years,respectively) and needs further observation.

According to Koenigsberg’s modified measurement technique, the basal angle in children ranged from 104° to 124°, with 95% confidence limits of the means for children 113°–115° [18]. Koenigsberg et al. did not specify the ages of “children” in their report. Our measurement showed the basal angle ranged from 111.9° to 134° with mean of 122.51°. The majority of our patients in this series were having imaging character of platybasia when compared with the normal mean published by the Koenigsberg et al. However, we are not certain about the clinical significance of wider basal skull base angle among our patients, but we suspect these differences are likely due to developing skull anatomy during infancy and early childhood.

## Conclusion

Suspecting Chiari I malformation among non-verbal children or those with limited vocabulary may pose a clinical challenge. Based on our experience, headaches/irritability and oropharyngeal/respiratory symptoms are their primary presenting symptoms.

Our experience analyzing 16 infants and toddlers indicates that the recurrence rate tends to be higher among the patients after Type 1 procedure, particularly those younger than 18 months, than after Type 2 procedure though there is no statistical difference. However, when the postoperative MRI indicates persistent crowdedness at the PFD site, the recurrence rates of Chiari I become significantly greater in either group. Duraplasty with dural graft offers better decompression by our observation. However, one should keep it mind that the occurrence of CSF-related complications, particularly pseudomeningocele, after duraplasty is high in this age group at primary or at redo (4 out of 11 cases). Meticulous water-tight dural closure needs to be stressed. Because of small number of the cases of surgically treated Chiari I malformation presented here, it is difficult to make a final conclusion as to which one being more safe and effective between the procedures in this particular group. Multicenter cooperative study would be helpful.
